# Analysis of risk factors for adverse outcomes in HIV/AIDS patients with ESRD undergoing hemodialysis: a cohort study

**DOI:** 10.3389/fneph.2026.1818921

**Published:** 2026-07-27

**Authors:** Guohai Wei, Zhenghong Huang, Lizhen Feng, Qing Wang, Zhenkun Zhang, Bo Chen

**Affiliations:** 1 Department of Blood Purification, Guangxi Zhuang Autonomous Region Chest Hospital, Liuzhou, Guangxi, China; 2 Department of Infectious Diseases, Guangxi Zhuang Autonomous Region Chest Hospital, Liuzhou, Guangxi, China; 3 Department of Science and Education, Guangxi Zhuang Autonomous Region Chest Hospital, Liuzhou, Guangxi, China

**Keywords:** adverse outcomes, concurrent infection, end-stage renal disease, hemodialysis, HIV/AIDS, risk factors

## Abstract

**Background:**

Infection is the second leading cause of death and hospitalization in hemodialysis (HD) patients. The occurrence of infections in HIV/AIDS patients with End-Stage Renal Disease (ESRD) undergoing maintenance hemodialysis is influenced by patient characteristics and related treatment regimens. Currently, there is limited data both domestically and internationally on the risk factor analysis for adverse outcome following infections in HIV/AIDS patients with ESRD receiving maintenance hemodialysis. This study aims to identify the risk factors associated with adverse outcomes following infections during hemodialysis in patients with HIV/AIDS and ESRD.

**Methods:**

In this retrospective cohort study, we evaluated the 9-month treatment outcomes of 70 HIV/AIDS patients with ESRD and concurrent infections undergoing maintenance hemodialysis at a hospital in Guangxi between January 2019 and December 2024. The patients were divided into adverse outcome group(defined as mortality or severe complications leading to prolonged hospitalization) and survival group. Data analysis was performed using IBM SPSS Statistics version 26.0, with statistical significance set at *P* ≤ 0.05, to identify adverse outcome risk factors for concurrent infections in HIV/AIDS patients with ESRD.

**Results:**

Among the 70 enrolled patients, the age ranged from 11 to 86 years. Adverse outcomes occurred in 48 cases (68.57%), while 22 patients (31.43%) survived without severe events. Multivariate analysis identified low-level hemoglobin(HB)(OR 0.952(95%CI 0.908,0.998),*P* = 0.042) and platelet (PLT)(OR 0.987(95%CI 0.975,1.000),*P* = 0.046).

**Conclusion:**

Adverse outcomes are common among HIV/AIDS patients with ESRD who develop infections during maintenance hemodialysis. Low hemoglobin and low platelet levels are significant risk factors for poor prognosis in this patient population. Early identification and management of these hematologic abnormalities may help improve clinical outcomes.

## 1 Introduction

**Figure 1:**
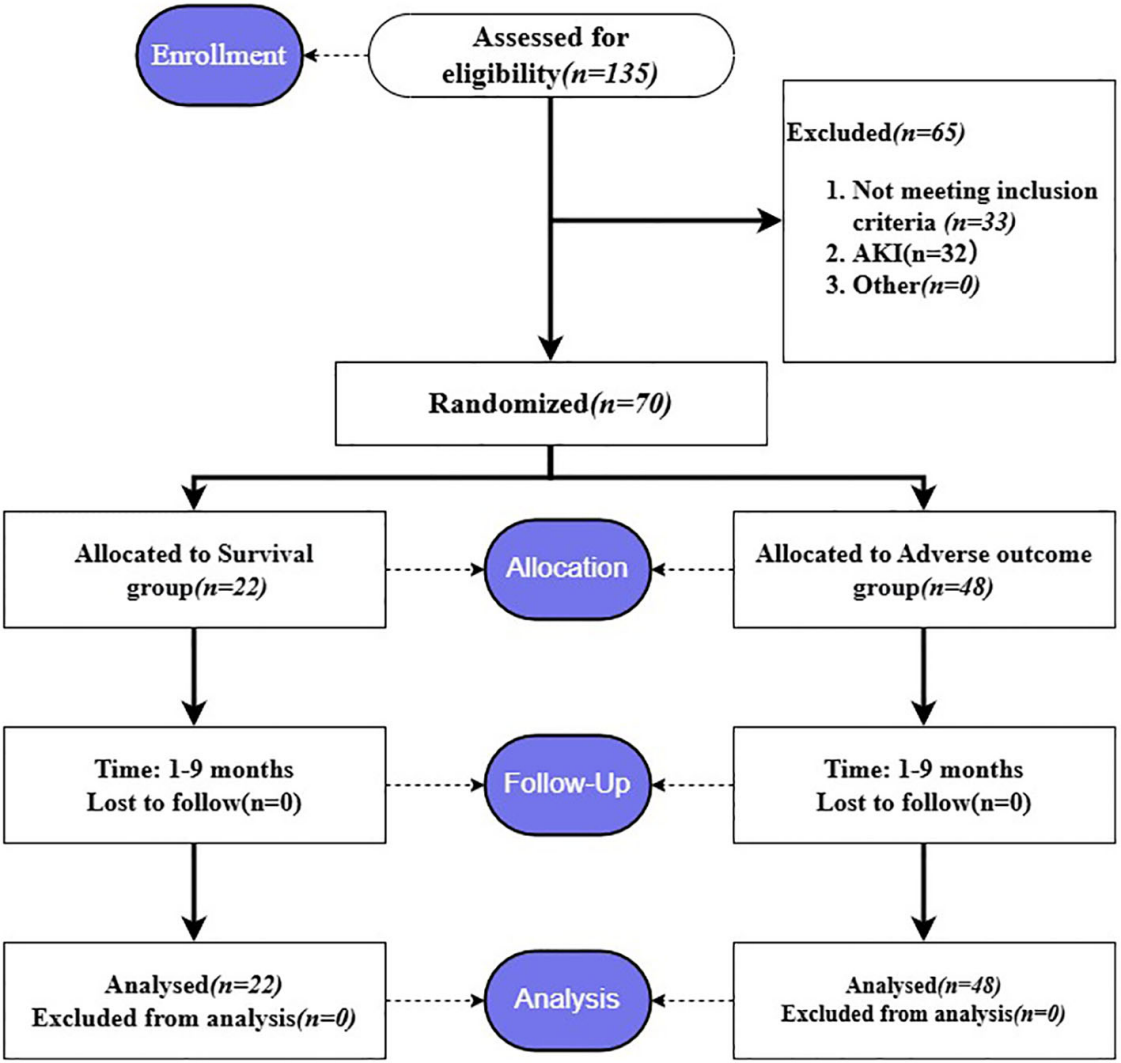
Participant flow chart.

Advances in Antiretroviral Therapy (ART) have transformed HIV/AIDS into a chronic condition that can be managed long-term. However, this progress has simultaneously led to an increased prevalence of non-AIDS-defining comorbidities, including end-stage renal disease (ESRD). Consequently, a growing number of patients require lifelong renal replacement therapy, with hemodialysis (HD) being the primary treatment modality. Dummy **Figure 1**.

It is reported that the infection rate in HD patients is more than 26 times higher than that in the general population (1), with specific pathogen infection rates being 100 to 200 times higher (2). These infections represent the second leading cause of hospitalization and mortality among dialysis populations (3–5). Both national and international guidelines, as well as national policy initiatives (6–9), recommend the use of arteriovenous fistulas (AVF) whenever possible, since patients using central venous catheters (CVC) face the highest risks of infections and other complications (3, 10, 11). Despite the risks associated with using CVCs, these devices remain the primary type of hemodialysis access for most HD patients in Ireland and internationally (12, 13).

The intersection of HIV/AIDS and ESRD forms a patient population with exceptionally complex clinical conditions, bearing the burden of a “triple-hit” syndrome: persistent immunodeficiency, uremia-related immune dysfunction, and repeated invasive procedures through vascular access. Hemodialysis itself constitutes a well-established risk factor for infections, ranging from vascular access-related bacteremia to bloodborne viral pathogens. When superimposed with HIV infection, these risks increase exponentially, leading to atypical infection presentations, a broader spectrum of pathogens (including opportunistic organisms), and complex pharmacological interactions between ART and antimicrobial agents. Despite the profound impact, robust clinical data on infection prevention and management for this specific intersectional population remain extremely limited.

Given that current practices primarily draw upon guidelines for the general hemodialysis population or individuals with HIV/AIDS, these are insufficient to address the unique vulnerabilities of this specific cohort. Therefore, this study aims to elucidate the epidemiological and clinical characteristics of hemodialysis-related infections in HIV/AIDS patients with coexisting ESRD and to identify independent risk factors for adverse outcomes. The findings are expected to inform the development of targeted clinical protocols, ultimately aimed at reducing morbidity and mortality in this high-risk population.

Currently, data on the risk factors for adverse outcomes following infections in HIV/AIDS patients with ESRD undergoing maintenance hemodialysis remain limited. Identifying these risk factors is essential for early risk stratification, guiding targeted interventions, and ultimately improving prognosis in this vulnerable population. Therefore, this retrospective cohort study aimed to investigate the independent risk factors for adverse outcomes following hemodialysis-related infections in HIV/AIDS patients with ESRD.

## 2 Materials and methods

### 2.1 Study design and participants

From January 2019 to December 2024, 70 HIV/AIDS patients with coexisting ESRD complicated by infection were enrolled. All subjects met the Chinese diagnostic criteria for sexually transmitted diseases and HIV/AIDS (10). The inclusion criteria (11) were as follows: (1) confirmed HIV/AIDS; (2) undergoing their first hemodialysis session; (3) diagnosed with chronic kidney disease (CKD) stage 5. Exclusion criteria included: (1) patients with a history of peritoneal dialysis or hemodialysis; (2) patients following kidney transplantation.

Definitions of outcomes.

Patients were categorized into two groups based on their 9-month follow-up outcomes:

Adverse outcome group: defined as all-cause mortality or severe complications (e.g., prolonged hospitalization, intensive care unit admission, or major adverse cardiovascular events) occurring during the follow-up period.

Survival group: defined as patients who remained alive without the occurrence of the above-mentioned severe complications during the follow-up period.

### 2.2 Clinical and laboratory data

1. General information. This includes sex, age, marital status, ethnicity, educational level, occupation, and geographical distribution.
2. Dialysis-related parameters. These include post-dialysis changes in body weight, systolic blood pressure, and diastolic blood pressure dialysis duration, ultrafiltration volume, dialysis access, and dialysis modality.
3. Infectious disease-related information. This includes HIV, HBV, HCV, and syphilis status; route of HIV/AIDS infection; whether antiretroviral therapy (ART) is being administered; adherence to AIDS treatment; and awareness of infection status.
4. Imaging findings. These include urinary system ultrasonography and echocardiography.
5. Clinical indicators. These include complete blood count (CBC) parameters (for instance WBC); arterial blood gas parameters (e.g., pH, partial pressure of oxygen); renal function parameters (e.g., glucose, urea); heart failure markers (e.g., N-terminal pro-B-type natriuretic peptide, NT-proBNT); electrolyte panel (five items, for instance potassium); liver function parameters (e.g., total bilirubin); nutritional indicators (e.g., total protein, TP); lipid profile parameters (e.g., total cholesterol); and coagulation parameters (e.g., prothrombin time).

### 2.3 Statistical analysis

Data are described as mean ± standard deviation (SD) for normally distributed variables, and as median and interquartile range (IQR) for asymmetrically distributed variables. Comparisons between groups were performed using unpaired t-tests for normally distributed data and non-parametric Mann-Whitney U tests for non-normally distributed data. All variables demonstrating a significant association in univariate analyses were evaluated for inclusion in a multivariate regression model to predict treatment outcomes in HIV/AIDS patients undergoing hemodialysis; unstandardized regression coefficients (B) and their 95% confidence intervals (CIs) are reported. All statistical analyses were performed using SPSS version 26.0, and a two-tailed *P* value of.05 was considered statistically significant.

## 3 Results

### 3.1 Treatment outcomes status

Between 2019 and 2024, dialysis information was collected from 70 patients at the dialysis center. After 9 months of regular dialysis, 22 patients (31.43%) survived without severe events, while 48 patients (68.57%) were in the adverse outcome group. See **Table 1** for details.

**Table 1:** Outcomes of infections following hemodialysis in HIV/AIDS patients with ESRD.

Time	Survival group (n,%)	Adverse outcome group (n,%)	Total
2019	2 (25.0)	6 (75.0)	8
2020	3 (23.08)	10 (76.92)	13
2021	3 (25.0)	9 (75.0)	12
2022	6 (31.58)	13 (68.42)	19
2023	3 (33.33)	6 (66.67)	9
2024	5 (55.56)	4 (44.44)	9
Total	22 (31.43)	48 (68.57)	70

### 3.2 Demographic characteristics

The patients’ ages ranged from 11 to 86 years. Among HIV/AIDS patients with coexisting ESRD who developed infections after hemodialysis, the largest proportion were male(52,74.29%). Furthermore, despite undergoing maintenance hemodialysis for up to nine months following the infection, the clinical condition remained severe, 34(65.4%) male patients still observed with treatment failure. See **Table 2** for details.

**Table 2:** Comparison of baseline characteristics between positive outcomes group and adverse outcomes group.

Parameters	Survival group (n,%)	Adverse outcome group (n,%)	*P* value
Gender			0.329
Male	18 (34.6)	34 (65.4)	
Female	4 (22.2)	14 (77.8)	
Age (y)			0.102
Youth	0 (2.7)	6 (15.4)	
Middle age	14 (54.1)	20 (35.4)	
Old age	8 (43.2)	22 (49.2)	
Marital status			0.174
Single	4 (16.2)	7 (12.3)	
Married	16 (70.3)	25 (60.0)	
Divorced	2 (10.8)	12 (20.0)	
Widowed	0 (2.7)	4 (7.7)	
Nation			0.115
Han nationality	16 (62.2)	31 (61.5)	
Zhuang nationality	6 (35.1)	9 (23.1)	
Other	0 (2.7)	8 (15.4)	
Educational status			0.485
Illiterate	3 (24.3)	12 (23.1)	
Primary school	116 (43.2)	26 (49.2)	
Secondary school	6 (27.0)	8 (23.1)	
Tertiary	2 (5.4)	2 (4.6)	
Occupation			0.741
Farmer	17 (86.5)	32 (69.2)	
Retire	2 (5.4)	5 (10.8)	
Unemployed	2 (5.4)	5 (7.7)	
Other	1 (2.7)	6 (12.3)	
Resident			0.876
Urban	4 (16.2)	8 (15.4)	
Rural	18 (83.8)	40 (84.6)	

### 3.3 Dialysis and infectious disease-related parameters

Parameters including post-dialysis changes in body weight and blood pressure, dialysis duration, ultrafiltration volume, dialysis access, and dialysis modality showed no significant impact on the treatment outcomes of HIV/AIDS patients with coexisting ESRD. The differences in outcomes between groups were not statistically significant (*P* > 0.05) (**Table 3**).

**Table 3:** Hemodialysis conditions.

Parameters	Survival group (n,%)/ (M,P_25_,P_75_)	Adverse outcome group (n,%)/ (M,P_25_,P_75_)	*P* value
Weight change after dialysis (kg)	0.0000 (0.0000,0.6500)	0.0000 (0.0000,0.8000)	0.476
Change in systolic blood pressure after dialysis (mmHg)	-3.0000 (-9.0000,1.0000)	-2.0000 (-9.5000,9.0000)	0.965
Change in diastolic blood pressure after dialysis (mmHg)	1.0000 (-3.5000,5.5000)	0.0000 (-4.0000,5.0000)	0.612
Time of hemodialysis (h)	2.0000 (2.0000,2.0000)	2.0000 (2.0000,2.0000)	0.692
Ultrafiltration rate (mL/h)	0.0000 (0.0000,650.0000)	0.0000 (0.0000,800.0000)	0.789
Dialysis pathway			0.495
Temporary catheter	22 (100.0)	47 (98.5)	
Long-term catheter	0 (0.0)	1 (1.5)	
Type of hemodialysis			0.495
HD	22 (100.0)	47 (96.9)	
CRTT	0 (0.0)	1 (3.1)	

No significant differences were observed between the adverse outcome and survival groups in terms of demographic characteristics, HIV transmission route, co-infections (HBV, HCV, syphilis), ART administration, treatment adherence, awareness of infection status, or CD4+ lymphocyte count (*P* > 0.05), but there were statistically significant differences between groups in ART use (*P* = 0.027<0.05) and treatment adherence (*P* = 0.035<0.05) (**Table 4**).

**Table 4:** Infectious disease conditions.

Parameters	Survival group (n,%)	Adverse outcome group (n,%)	P value
HIV			Na
Positive	22 (100.0)	48 (100.0)	
Negative	0 (0.00)	0 (0.00)	
HBV			0.275
Positive (HBsAg positive)	5 (22.2)	6 (13.8)	
Negative	17 (77.8)	42 (86.2)	
HCV			0.415
Positive	1 (5.4)	5 (7.7)	
Negative	21 (94.6)	43 (92.3)	
Syphilis			0.142
Positive	7 (32.4)	15 (27.7)	
Negative	15 (34.7)	33 (65.3)	
Routes of HIV transmission			0.584
Sexual transmission	22 (97.3)	44 (93.8)	
Bloodborne transmission	0 (2.7)	2 (3.2)	
Mother-to-child transmission	0 (0.0)	1 (1.5)	
Other	0 (0.0)	1 (1.5)	
On antiretroviral therapy			0.027
Yes	36 (97.3)	56 (86.2)	
No	0 (0.0)	10 (13.8)	
Adherence to HIV treatment			0.035
Yes	32 (86.5)	45 (69.2)	
No	4 (10.8)	21 (30.8)	
Awareness of HIV status			0.694
Yes	19 (91.9)	43 (84.6)	
No	3 (8.1)	5 (15.4)	
CD4+ lymphocytes (109/L)	19 (27.1)	46 (65.7)	0.977

### 3.4 Clinical laboratory parameters

Clinical laboratory parameters are critical determinants of patient treatment outcomes. Imaging findings indicated that abnormalities in the urinary and cardiac systems had no significant impact on the risk of post-hemodialysis infections in HIV/AIDS patients with coexisting ESRD (*P* > 0.05) (**Table 5**).

**Table 5:** Imaging findings.

Parameters	Survival group (n,%)	Adverse outcome group (n,%)	*P* value
Urinary system ultrasound			0.179
Normal	3 (29.7)	6 (18.5)	
Abnormal	17 (62.2)	36 (72.3)	
Not done	2 (8.1)	6 (9.2)	
Echocardiogram			0.402
Normal	4 (16.2)	6 (13.8)	
Abnormal	14 (67.6)	33 (72.3)	
Not done	4 (16.2)	9 (13.8)	

No statistically significant differences were observed between the two groups in baseline parameters including WBC, C-reactive protein, procalcitonin, pH, partial pressure of oxygen, partial pressure of carbon dioxide, whole blood base excess, extracellular fluid base excess, oxygen saturation, oxygenation index, glucose, urea, creatinine, and uric acid (*P* > 0.05). However, statistically significant differences were found in nutritional indicators such as RBC (*P* = 0.039), HB (*P* = 0.001), hematocrit (*P* = 0.002), and PLT (*P* = 0.029); renal function indicators including NT-proBNP (*P* = 0.009); electrolyte parameters including K^+^ (*P* = 0.019) and Ca^+^ (*P* = 0.039); protein metabolism indicators including TP (*P* = 0.003); and DBil (*P* = 0.035), all exhibited statistically significant differences(P<0.05) (**Table 6**).

**Table 6:** Clinical indicators and related conditions.

Parameters	Survival group (M,P_25_,P_75_)	Adverse outcome group (M,P_25_,P_75_)	*P* value
WBC (×10^9^/L)	6.4700 (4.2500,10.3300)	7.1200 (5.2450,9.0450)	0.400
RBC (×10^12^/L)	2.7600 (2.3450,3.4300)	2.5000 (2.0300,3.0400)	0.039
HB (g/L)	88.0000 (74.5000,106.000)	74.0000 (63.0000,92.5000)	0.001
Hematocrit (%)	26.7000 (21.7000,31.2000)	21.8000 (19.2000,25.7500)	0.002
PLT (×10^9^/L)	210.000 (139.000,339.5000)	169.0000 (105.000,250.5000)	0.029
C-RCP (mg/L)	37.1000 (14.2000,131.000)	35.8000 (12.000,106.7000)	0.057
PCT (ng/mL)	0.7600 (0.2675,3.2425)	1.1200 (0.3800,3.0700)	0.785
PH	7.28000 (7.2000,7.35000)	7.2800 (7.1800,7.3800)	0.887
PaO2 (mmHg)	108.5000 (91.5000,135.5000)	102.000 (86.000,130.000)	0.720
PaCO_2_ (mmHg)	25.000 (20.2500,28.7500)	25.000 (19.0000,29.0000)	0.720
BE-B (mmol/L)	-12.8500 (-17.2500,-9.3250)	-13.4000 (-18.2500,-7.0000)	0.890
BE-ECF (mmol/L)	-14.6000 (-18.9250,-10.8000)	-15.000 (-20.0500,-7.9000)	0.788
SaO_2_/SpO_2_ (%)	98.000 (96.000,99.0000)	97.0000 (96.0000,98.0000)	0.977
PaO_2_/FiO_2_ (mmHg)	476.000 (419.5000,605.000)	467.0000 (386.0000,567.0000)	0.586
Glu (mmol/L)	6.9000 (5.5000,8.9575)	6.2400 (5.1700,8.0350)	
Urea (mg/dL)	25.9000 (17.1000,33.5500)	25.9000 (22.7000,39.5500)	0.066
Cr (μmol/L)	782.000 (505.5000,961.5000)	873.0000 (576.5000,1207.0000)	0.092
UA (μmol/L)	615.5000 (407.0000,738.8500)	519.0000 (463.0000,746.5000)	0.788
β2-MG (μg/mL)	14.1300 (13.3900,0.000)	14.4800 (9.9525,25.9150)	1.000
Hcy (μmol/L)	25.2000 (15.8000,30.4000)	28.8000 (20.5750,47.7000)	0.202
Cys-C (mg/L)	4.4500 (3.2900,5.3300)	5.0900 (4.1700,6.1775)	0.253
PA (mg/L)	181.000 (109.0000,244.0000)	175.0000 (106.0000,255.0000)	0.617
CrCl (mL/min)	13.4200 (10.7575,20.0750)	11.5600 (8.6500,14.7650)	0.663
NT-proBNP (ng/L)	200.000 (200.000,600.000)	500.000 (200.000,3000.0000)	0.009
K^+^ (mmol/L)	3.7300 (3.5450,4.6050)	4.5000 (3.6500,5.2750)	0.019
Na^+^ (mmol/L)	134.0000 (130.000,136.000)	135.0000 (130.0000,139.0000)	0.494
Cl^-^ (mmol/L)	105.000 (100.4500,110.500)	104.9000 (100.0000,110.0000)	0.985
Ca^2+^ (mmol/L)	2.1000 (1.8600,2.2200)	1.9300 (1.7650,2.0600)	0.039
Mg^2+^ (mmol/L)	0.8100 (0.7425,0.9400)	0.8600 (0.7300,0.9850)	0.875
CO_2_ (mmol/L)	13.5500 (9.5000,19.3750)	13.6000 (8.4000, 18.000)	0.730
P (mmol/L)	1.5500 (1.0950,2.16000	1.9400 (1.3350, 2.7450)	0.683
Fe (μmol/L)	10.6000 (5.8400, 19.7000)	17.1000 (4.8450,22.9750)	0.683
TBil (μmol/L)	5.0200 (3.3000, 10.9450)	3.9900 (2.7500,7.4350)	0.121
DBil (μmol/L)	2.6200 (1.6350,6.8850)	1.7600 (0.9300,4.1150)	0.025
IBil (μmol/L)	2.4700 (1.7950,4.3900)	2.5100 (1.5800,3.6550)	0.277
ALT (U/L)	18.0000 (11.000,32.5000)	19.0000 (10.5000,36.000)	0.785
AST (U/L)	29.0000 (19.5000,51.000)	27.0000 (17.5000,52.0000)	0.761
ALP (U/L)	133.000 (96.5000,177.2500)	128.0000 (80.5000,163.7500)	0.257
TP (g/L)	79.4000 (64.3000,86.5500)	66.9000 (60.9800,75.30000	0.003
ALB (g/L)	33.000 (27.8500,37.4500)	28.8000 (23.8500,32.7000)	0.134
TC (mmol/L)	3.3600 (2.9800,4.3850)	3.7850 (2.9250,4.4075)	0.400
TG (mmol/L)	1.9300 (1.1050,2.6300)	1.7150 (1.1875,2.9600)	0.494
HDL-C (mmol/L)	0.8600 (0.5900,1.0400)	1.0000 (0.7550,1.2375)	0.331
LDL-C (mmol/L)	1.9500 (1.5200,2.5300)	2.1050 (1.2825,2.9275)	0.441
PT (s)	13.1500 (12.0000,14.6250)	13.1000 (11.7750,14.2000)	0.292
INR	1.0900 (0.9925,1.2200)	1.0700 (0.9875,1.1700)	0.203
APTT (s)	33.6000 (24.7500,37.8000)	35.1000 (27.2750,42.3750)	0.541
TT (s)	18.6000 (16.9750,19.5750)	18.5000 (17.3000,19.6000)	0.832
FIB (g/L)	4.4600 (3.1975,5.7225)	4.4000 (3.3150,5.6550)	0.405
D-dimer (mg/L)	1.4700 (0.5600,4.5750)	2.0900 (1.4575,4.01000	0.230

PCT, Procalcitonin; PaO_2_, Partial pressure of oxygen; PaCO_2_, Partial pressure of carbon dioxide; BE-B, Base excess in blood; BE-ECF, Base excess in extracellular fluid; SaO_2_/SpO_2_, Oxygen saturation; PaO_2_/FiO_2_, Oxygenation index; Glu, Glucose; BUN, Blood urea nitrogen; Cr, Creatinine; UA,Uric acid; β2-MG, Beta-2 microglobulin; Hcy, Homocysteine; Cys-C, Cystatin C; PA, Prealbumin; CrCl, Creatinine clearance rate; NT-proBNP, N-terminal pro-B-type natriuretic peptide; K^+^, Potassium; Na^+^, Sodium; Cl^-^, Chloride; Ca^2+^, Calcium; Mg^2+^, Magnesium; CO_2_, Carbon dioxide; P, Phosphorus; Fe, Iron; TBil, Total bilirubin; DBil, Direct bilirubin; IBil, Indirect bilirubin; TC, Total cholesterol; TG, Triglycerides; HDL-C, High-density lipoprotein cholesterol; LDL-C, Low-density lipoprotein cholesterol; PT, Prothrombin time; INR, International normalized ratio;APTT, Activated partial thromboplastin time;TT, Thrombin time;FIB, Fibrinogen.

### 3.5 Risk factors

**Figure 2:**
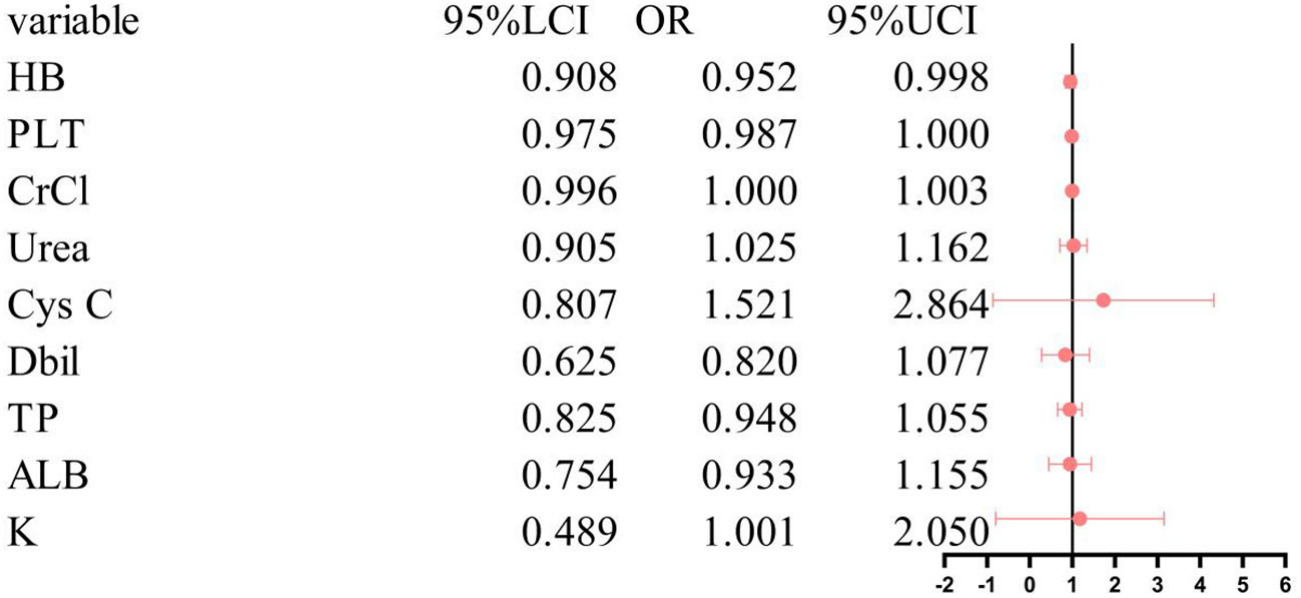
Forest plot.

Subsequently, we conducted a further analysis of all variables. The linearity test results indicated that the variance inflation factor (VIF) values for all independent variables ranged from 1.12 to 2.58 (see **Table 7**), which are significantly below the critical threshold of 5 (or 10). Therefore, it can be concluded that the model does not suffer from severe multicollinearity, and the coefficient estimates for each independent variable in the subsequent regression analysis are stable and reliable. Given that the number of variables included in the regression analysis is generally 10% of the total enrolled participants, we incorporated both significant and potential variables into the analysis. Variables and assignments see **Table 8**. After adjusting for multiple factors in the logistic regression analysis, two variables remained significant in the model: low hemoglobin level (OR 0.952, 95% CI 0.908–0.998, *P* = 0.042) and low platelet count (OR 0.987, 95% CI 0.975–1.000, *P* = 0.046) were identified as statistically significant predictors of adverse outcomes in HIV/AIDS patients with ESRD who developed infections during maintenance hemodialysis (**Table 9**, **Figure 2**).

**Table 7:** Collinearity test.

Parameters	Unstandardized coefficients (B)		Standardized coefficients (Beta)	t	*P* value	Collinearity statistics	
	B	Standard error	Beta			Tolerance	VIF
Constant	2.418	0.521		4.644	0.000		
HB	-0.006	0.002	-0.312	-2.916	0.005	0.826	1.210
PLT	-0.001	0.001	-0.234	-2.245	0.028	0.874	1.144
CrCl	-0.008	0.012	-0.125	-.647	0.520	0.253	3.953
Urea	0.001	0.001	0.057	0.497	0.621	0.728	1.373
Cys-C	0.039	0.065	0.117	0.598	0.551	0.249	4.013
DBil	0.001	0.003	0.060	0.496	0.621	0.659	1.518
K^+^	0.034	0.036	0.097	0.937	0.352	0.887	1.128
Ca^2+^	0.022	0.026	0.086	0.864	0.391	0.953	1.050
TP	-0.001	0.001	-0.173	-1.761	0.083	0.985	1.015
ALB	-0.007	0.003	-0.240	-2.374	0.020	0.929	1.077

**Table 8:** Variables and assignments.

Parameters	Assignment
Treatment Outcomes	0=Survival group, 1=Adverse outcome group

**Table 9:** Multivariate analysis.

Parameters	OR (95%CI)	*P* value
HB	0.952 (0.908,0.998)	0.042
PLT	0.987 (0.975,1.000)	0.046
CrCl	1.000 (0.996,1.003)	0.834
Urea	1.025 (0.905,1.162)	0.693
Cys-C	1.521 (0.807,2.864)	0.195
DBil	0.820 (0.625,1.077)	0.154
TP	0.948 (0.852,1.055)	0.330
ALB	0.933 (0.754,1.155)	0.525
K^+^	1.001 (0.489,2.050)	0.998
Ca^2+^	6.92 (0.162,296.04)	0.313

## 4 Discussion

Hemodialysis, as a critical life-sustaining intervention for chronic kidney disease (CKD), effectively replaces renal function by removing metabolic wastes and maintaining fluid homeostasis, thereby reducing the risk of co-infection in HIV/AIDS patients with ESRD undergoing hemodialysis.

The study suggests that abnormal platelet function is the primary cause of bleeding symptoms and prolonged bleeding time in patients with end-stage renal disease (ESKD) (14). Studies have demonstrated that up to 50% of patients with ESKD are affected by hemostatic dysfunctions, with manifestations that range from easy bruising and prolonged bleeding at the vascular access site to potentially life-threatening conditions, such as gastrointestinal and intracranial bleeding (15, 16). Platelet dysfunction may be the result of decreased dense granule content, decreased sensitivity to platelet agonists, abnormal expression of platelet glycoproteins, defective arachidonate metabolism and depressed prostaglandin metabolism as well as impaired platelet adhesiveness. Platelet dysfunction is thought to be caused by the action of uremic toxins, anemia, increased nitric oxide production, the use of medication like aspirin etc. (16–19). The improvement of uremic thrombocytopathies by HD techniques indicates uremic toxins are, at least partially, responsible for the observed platelet dysfunctions (20). Similarly, this study found that PLT levels significantly influence the treatment outcomes of patients with HIV/AIDS and end-stage renal disease (ESRD) who develop infections after hemodialysis; specifically, platelet count (OR 0.987 [95% CI 0.975–1.000], *P* = 0.046) was identified as an independent risk factor for adverse outcomes in these patients.

The prevalence of anemia in patients with chronic kidney disease ranges from 7% to 86% (21–23). This wide variation can be partially attributed to the different criteria used to define anemia, as well as the heterogeneity of the study populations, encompassing both pre-dialysis patients and those undergoing various forms of renal replacement therapy. In dialysis patients, in contrast to the World Health Organization definition, anemia is characterized by HB levels falling below the recommended treatment target.

According to the WHO definition, the prevalence of anemia (defined by hemoglobin levels) in our sample of HIV/AIDS patients with ESRD was 97.14%, with mild anemia accounting for 5.71%, moderate anemia for 32.86%, severe anemia for 41.43%, and extremely severe anemia for 17.14%. This indicates a very high prevalence of anemia, despite all patients receiving erythropoietin therapy at the time of laboratory assessment. Comparisons with other studies reveal substantial variations. A cross-sectional study from Brazil, which included 120 patients undergoing peritoneal dialysis (PD), reported an anemia prevalence of 28%, with 87.5% of these patients receiving erythropoietin therapy (24). In contrast, a three-month retrospective study from Spain found that 89% of 343 PD patients had anemia, while 72.9% were receiving erythropoietin treatment (25). These discrepancies likely reflect heterogeneity across different populations in terms of study design, anemia definition, target hemoglobin levels, dialysis adequacy, iron status, and local treatment protocols. Nevertheless, all cited studies, including our own, confirm that anemia is a highly common complication in dialysis patients. Our study further confirms that low hemoglobin levels (OR 0.952, 95% CI 0.908,0.998, *P* = 0.042) are a risk factor for infection following hemodialysis in HIV/AIDS patients with ESRD.

In summary, anemia is a severe complication in HIV/AIDS patients with ESRD who develop co-infections after undergoing hemodialysis, with mild to moderate anemia being the predominant presentation. HB and PLT are independently associated with adverse outcomes following infections in HIV/AIDS patients with ESRD. Special attention should be given to the anemic status of HIV/AIDS patients with ESRD undergoing hemodialysis, and potentially to mitigate the risk of adverse outcomes following infections.

This study has several limitations that should be acknowledged. First, selection bias may exist simply due to the retrospective nature of our single-center study. Therefore, it is necessary to validate these findings through prospective investigations in multiple centers. Of note, our study implemented several measures to minimize such bias: (1) all laboratory assessments were conducted using a predetermined randomized design to minimize measurement bias; (2) eligible HIV/AIDS patients with ESRD and co-infection were enrolled as comprehensively as possible to reduce selection bias; and (3) data were collected as thoroughly as possible and analyzed using appropriate statistical methods. Second, this study did not address multiple potential confounding factors, such as medication history, dietary and exercise patterns, and patients’ psychological status, all of which should be considered to enhance the validity of the results. Third, although 70 patients were included in the data analysis, Due to limitations imposed by healthcare insurance policies, this study did not investigate the types of co-infections occurring in HIV/AIDS patients with end-stage renal disease (ESRD) undergoing hemodialysis, nor the completeness of infection-related data. Future research in larger, multicenter cohorts is needed to validate prognostic risk factors for different complications across various ethnic groups or comorbidity populations. Also, the viral load, and specific ART regimen information were not available in our database. These are important prognostic factors for outcomes in infected patients. Future prospective studies should collect these parameters for more comprehensive analyses. It should be noted that this study did not establish a non-infected control group, thus making it impossible to identify the risk factors underlying the transition from “non-infected” to “infected.” It is recommended that future studies design prospective cohorts incorporating non-infected individuals to comprehensively evaluate the two-stage risk factors for infection occurrence and adverse outcomes post-infection. Finally, in future work, we plan to conduct prospective studies to comprehensively further evaluate factors influencing survival rates in HIV/AIDS patients with ESRD with different characteristics, thereby proactively collecting longitudinal data on nutritional status and inflammatory markers.

## 5 Conclusion

Infection remains a significant complication, particularly in contemporary cohorts of HIV/AIDS patients with ESRD undergoing maintenance hemodialysis. Low levels of HB and PLT are significant risk factors, and associated infections impose a substantial burden on both patients and the healthcare system. Active surveillance for infections, coupled with quality improvement initiatives, should be an integral component of all dialysis programs to reduce dialysis-related infections and improve prognosis in HIV/AIDS patients with ESRD.

## Data Availability

The raw data supporting the conclusions of this article will be made available by the authors, without undue reservation.
